# Bayesian accuracy estimates for diagnostic tests to detect tuberculosis in captive sun bears (*Helarctos malayanus*) and Asiatic black bears (*Ursus thibetanus*) in Cambodia and Vietnam

**DOI:** 10.1371/journal.pone.0313007

**Published:** 2024-11-13

**Authors:** Kirsty Officer, Juan Carlos Arango-Sabogal, Simon Dufour, Konstantin P. Lyashchenko, Jonathan Cracknell, Shaun Thomson, Sokleaph Cheng, Kris Warren, Bethany Jackson

**Affiliations:** 1 School of Veterinary Medicine, Murdoch University, Perth, WA, Australia; 2 Free the Bears, Phnom Penh, Cambodia; 3 Department of Pathology and Microbiology, Faculty of Veterinary Medicine, Université de Montréal, Saint-Hyacinthe, Québec, Canada; 4 Chembio Diagnostics, Inc., Medford, New York, United States of America; 5 Knowsley Safari, Prescot, Merseyside, United Kingdom; 6 Animals Asia Vietnam Bear Rescue Centre, Vinh Phuc, Vietnam; 7 Medical Biology Laboratory, Institut Pasteur du Cambodge, Phnom Penh, Cambodia; 8 Centre for Terrestrial Ecosystem Science and Sustainability, Harry Butler Institute, Murdoch University, Perth, WA, Australia; Sungkyunkwan University - Suwon Campus: Sungkyunkwan University - Natural Sciences Campus, REPUBLIC OF KOREA

## Abstract

Effective control of tuberculosis (TB) depends on early diagnosis of disease, yet available tests are unable to perfectly detect infected individuals. In novel hosts diagnostic testing methods for TB are extrapolated from other species, with unknown accuracy. The primary challenge to evaluating the accuracy of TB tests is the lack of a perfect reference test. Here we use a Bayesian latent class analysis approach to evaluate five tests available for ante-mortem detection of pulmonary TB in captive sun bears and Asiatic black bears in Southeast Asia. Using retrospective results from screening of 344 bears at three rescue centres, we estimate accuracy parameters for thoracic radiography, a serological assay (DPP VetTB), and three microbiological tests (microscopy, PCR (Xpert MTB/RIF, Xpert MTB/RIF Ultra), mycobacterial culture) performed on bronchoalveolar lavage samples. While confirming the high specificities (≥ 0.99) of the three microbiological tests, our model demonstrated their sub-optimal sensitivities (<0.7). Thoracic radiography was the only diagnostic method with sensitivity (0.95, 95% BCI: 0.76, 0.998) and specificity (0.95, 95% BCI: 0.91, 0.98) estimated above 0.9. We recommend caution when interpreting DPP VetTB results, with the increased sensitivity resulting from treatment of weakly visible reactions as positive accompanied by a drop in specificity, and we illustrate how the diagnostic value of weak DPP VetTB reactions is particularly reduced if disease prevalence and/or clinical suspicion is low. Conversely, the reduced utility of negative microbiological tests on bronchoalveolar lavage fluid samples when prevalence and/or clinical suspicion is high is demonstrated. Taken together our results suggest multiple tests should be applied and accompanied by consideration of the testing context, to minimise the consequences of misclassification of disease status of bears at risk of TB in sanctuary settings.

## Introduction

Tuberculosis (TB) is an infectious airborne disease affecting humans, livestock, and wildlife worldwide. The disease results from infection with members of the *Mycobacterium tuberculosis* complex (MTBC), a small group of genetically similar TB-causing mycobacteria [1]. Human TB is primarily caused by *Mycobacterium tuberculosis* and remains a substantial global public health challenge [2], whilst TB in livestock, caused by *Mycobacterium bovis*, impacts production, livelihoods, and trade, despite decades of management efforts [3, 4]. The transmission of human strains of *M*. *tuberculosis* to wildlife hosts, particularly elephants, is an emerging concern [5–7], and has occurred in sun bears (*Helarctos malayanus*) and an Asiatic black bear (*Ursus thibetanus*) in Cambodia [8], and sloth bears (*Melursus ursinus*) in India [9]. These findings highlight the need for surveillance of this ostensibly human pathogen across an expanding range of host species.

The availability and suboptimal accuracy of diagnostic tests are barriers to the successful control of TB across all host species [10–12]. For incidental or uncommon hosts, the feasibility of developing and validating species-specific tests is further limited by marketing and cost-benefit considerations. The identification of humans infected with *M*. *tuberculosis* is complicated by the range of possible outcomes following infection, including primary progression to active, transmissible, disease, or suppression of mycobacterial replication by the immune system along a spectrum of non-infectious latent, quiescent, or incipient disease states which may progress to active disease in the future [13–16]. Transition from the non-infectious state to active disease is poorly defined, and may or may not be accompanied by clinical signs, however prompt diagnosis and treatment before invasion of the lungs, aerosolisation, and onward transmission inevitably occurs is key to effective outbreak management [17]. The progression of TB along a similar diversity of disease states is likely yet not widely described in other mammalian species [18, 19], with this lack of clarity adding to the challenge of interpreting diagnostic test results in exposed animals.

TB diagnostic methods either directly detect mycobacteria or indirectly detect responses to infection. Commonly used direct methods include microscopy to visualise acid-fast bacteria, molecular tests for mycobacterial DNA, and culture of mycobacteria. A positive culture confirms TB [20, 21], however, when used ante-mortem relies on recovery of mycobacteria from the airways, using methods such sputum production, bronchoalveolar lavage, or, in elephants, the trunk wash technique [22, 23]. Indirect methods include imaging for characteristic pathological changes, and tests for immunological responses. Thoracic radiography is widely used for human TB screening [24, 25], although differentiation of subtle changes from those caused by other respiratory conditions is a recognised challenge [26, 27]. Immunological tests can detect responses to mycobacterial antigens before disease is active, making them particularly appealing in wildlife species where there are practical constraints on direct sampling and imaging [11]. While tests for cell mediated-immunity, such as the tuberculin skin test and interferon gamma-release assays, are used to detect a response to mycobacterial infection in primates and ruminants [28, 29], species specificity, cross reactions, and practical limitations due to thick or haired skin mean they are not commonly used in other species. Despite their convenience, serological tests for humoral immunity are not currently recommended for the diagnosis of TB in humans due to variable and inconsistent antibody responses [30, 31], and in cattle seropositivity is associated with advancing active disease [32]. Serodiagnostics have however shown promise in certain hosts, with consistent and robust responses to key MTBC antigens demonstrated in elephants and cervid species before active disease was detected by other means [33, 34]. A subsequently developed serological assay (Dual Path Platform (DPP) VetTB, Chembio Diagnostics Inc., Medford NY, USA) has shown high accuracy in these target species [34, 35], however its reliability is variable in other hosts [36–41]. Given the possibility that immunodominant antigens may be species-specific [42], the accuracy of the DPP VetTB assay when applied off-label to newly reported hosts, such as bears, warrants evaluation.

Hundreds of sun bears and Asiatic black bears live in sanctuaries in Cambodia and Vietnam following confiscation by or surrender to government authorities in efforts to combat illegal wildlife trade. Both countries have high human TB burdens [2], and human contact is thought to underly the emergence of TB in bear sanctuaries in the region [8]. A core consideration for disease control in bear sanctuaries is the selection of appropriate tests to accurately determine the true TB status of resident and incoming bears. The lack of a suitable ante-mortem reference test to enable detection of the range of possible TB disease states limits the use of standard methods to evaluate diagnostic test accuracy. A Bayesian latent class analysis (BLCA) approach addresses this by allowing inferences to be drawn on test accuracy in the absence of a perfect reference test [43]. The true infection status of sampled individuals is treated as unobserved, or “latent”, with each individual assumed to have a probability of infection based on the combination of prior knowledge and observed test outcomes [44]. Given this, we aimed to use a BLCA approach to estimate the diagnostic accuracy of five locally accessible tests (microscopy, PCR, and culture of respiratory tract samples, thoracic radiography, and the DPP VetTB serological assay) for the diagnosis of TB in sun bears and Asiatic black bears. Secondary aims included assessing the impact of the test positive threshold on the accuracy of the serological test, and using predictive values and likelihood ratios to describe the practical application of tests under different clinical scenarios.

## Materials and methods

This article was written following the outline for Standards for Reporting of Diagnostic accuracy studies that use Bayesian Latent Class Models (BLCMs) [45].

### Study design

This retrospective study uses routine health monitoring data from bears at three sanctuaries with TB infection risks. The target population is sun bears or Asiatic black bears in captivity with potential exposure to *M*. *tuberculosis*. The source population included sun bears and Asiatic black bears at sanctuaries located in Cambodia (Site 1: Cambodia Bear Sanctuary) and Vietnam (Site 2: Vietnam Bear Rescue Centre; Site 3: Cat Tien Bear Sanctuary). Bears arrive at these facilities after voluntary transfer or interception by government authorities following removal from the wild and/or illegal captivity. The duration and nature of the interim captivity between capture from the wild and arriving at the sanctuaries is variable, ranging from days to years. Over the study period (February 2016 to March 2023) the total numbers of bears at risk at each site were dynamic, with individuals entering and leaving due to new arrivals, deaths, and transfers (Table 1). The number of infected bears in the source population was unknown, however at least one case of TB had historically been reported at each site. At sites 1 and 2, *M*. *tuberculosis* was confirmed as the causative agent in 32 and 1 case respectively, and at the third site a case of TB was diagnosed post-mortem on gross pathology and histopathology, however the species of mycobacteria was not confirmed. No treatment for TB has been carried out at any of the sites. To be eligible for inclusion in the study, bears were sun bears or Asiatic black bears at any of the three sites with a set of relevant test results available within the study period.

**Table 1:** Cumulative bear populations at risk by species and study location between February 2016 and March 2023.

Study site	Name	Country	Study site coordinates	Total bears at risk (range^a^)	Sun bears (range^a^)	Asiatic black bears (range^a^)
**1**	Cambodia Bear Sanctuary	Cambodia	11°18’00.9"N 104°48’04.9"E	162 (117–135)	111 (76–97)	51 (37–45)
**2**	Vietnam Bear Rescue Centre	Vietnam	21°25’41.1414"N 105°37’36.6306"E	261 (161–219)	14 (10–14)	247 (151–205)
**3**	Cat Tien Bear Sanctuary	Vietnam	11°24’ 30.0702"N 107°24’ 50.4246"E	55 (35–50)	13 (9–13)	42 (26–37)

^a^range = range of population at risk at any time point during the study period

We used data generated through health monitoring of bears at all three sites (convenience sampling) and included bears with and without clinical signs consistent with TB at the time of testing. One set of results generated from a single testing point per bear was included. If a bear had multiple testing points within the study period, only results from the most recent testing point were included. Not all historical TB cases were eligible due to not having a full set of test results, however any bear with culture-confirmed TB and a concurrent (within one month) DPP VetTB result available was included in a separate descriptive analysis of serological test line reactivity.

### Ethics statement

This study opportunistically and retrospectively used diagnostic test results data generated during routine veterinary and post-mortem procedures. No animals were anaesthetised or euthanised for the specific purpose of this study, and all testing was carried out using standard veterinary techniques for clinical examination, disease screening, and diagnostic investigation. Ethics approval for the use of animal testing data in this study was obtained from the Murdoch University Animal Ethics Committee, under permit number R3276/20. Where relevant, this study is reported in accordance with ARRIVE guidelines (https://arriveguidelines.org/).

### Test methods

#### Sample collection

All sample collection was performed under general anaesthesia. Bears were immobilised via intramuscular injection delivered by blow dart or injection pole using a zolazepam and tiletamine combination (1.25 mg/kg) reconstituted with medetomidine (0.0125 mg/kg). After intubation anaesthesia was maintained with isoflurane and oxygen delivered via a circle re-breathing circuit. Blood samples were collected via jugular venepuncture into sterile plain serum collection tubes. After standing for at least 10 min at room temperature, blood tubes were centrifuged at 1500–3000 rpm for 10 min, and serum pipetted into a sterile labelled cryovial. Serum was frozen at—20°C if testing was not immediate. Bronchoalveolar lavage (BAL) samples were collected by passing a sterile 10–12 French 125 cm feeding tube through a previously placed endotracheal tube until resistance was felt, indicating the level of the lower airways (usually 70–90 cm depending on the size of the bear). A volume of 60–200 ml of sterile saline was introduced to the airways using a 60 ml syringe attached to the tube, followed by re-aspiration while an assistant provided gentle rolling of the bear’s chest. The recovered fluid was placed into a sterile labelled collection vial, refrigerated, and transported to the respective laboratory within 48 hr.

#### Serological test

Serological testing was performed in-house at each site using a commercially available assay kit for the detection of antibodies to *M*. *tuberculosis* and *Mycobacterium bovis* in serum, plasma or whole blood from elephants, and *M*. *bovis* in serum from deer. The DPP VetTB rapid immunochromatographic test is designed to detect independent IgG antibody responses against two key mycobacterial antigens; the MPB83 protein and the CFP10/ESAT-6 fusion protein [34, 35]. The test was carried out according to the manufacturer’s instructions using 5 μl of fresh or thawed serum. Antibody responses are represented at test Line 1, indicating a measurable response to MPB83 and suggesting the sample is reactive for TB (caused by members of the MTBC) or mycobacteria other than TB (MOTT), and test Line 2, indicating a measurable response to CFP10/ESAT-6 and suggesting the sample is reactive for TB. Responses to both lines on the same test suggest the sample is reactive for TB. According to the manufacturer’s instructions, intensities of the test lines may vary, and test lines are considered reactive regardless of intensity. The presence of test lines was read by eye and lines were classified based on their relative visible intensity. Reading took place immediately on test completion, under consistent lighting conditions. Tests were valid if there was a clearly visible line in the control position. Reactive test lines were classified as strong if the line was clearly visible, complete, and of a strength comparable to the control line, whereas faint to barely visible lines were classified as weak. Tests were classified as negative if there were no visible lines at either of the test line positions. Regardless of the test operator, the result was confirmed by the attending veterinarian, who had knowledge of the history and clinical signs. Two case definitions for the serological test were used in subsequent statistical analyses. First, only tests with a strong line at one or both test positions were treated as positive. Second, tests with a weak or strong line at one or both test positions were considered positive.

#### Thoracic radiography

Thoracic radiographs were taken by veterinary staff using available equipment; either computed or digital image detection and viewing systems. At least three radiographic views of the thorax were taken including left lateral, right lateral and ventrodorsal views. Images were taken on full inspiration, with assisted positive pressure ventilation if needed. Images were viewed by the attending veterinarian at each site with knowledge of the clinical history, and findings were noted in the bear’s clinical record. These clinical records were then reviewed by the lead author (KO) in order to classify the radiographic interpretations. Studies were classified as positive if terms used to describe observed changes were consistent with those expected in cases of pulmonary TB, including mentions of: consolidation, increased opacity, calcification, pneumonia, granuloma, pleural fluid, thoracic lymph node enlargement, and/or if TB was mentioned as a differential diagnosis.

#### Microbiological tests

Microbiological testing was carried out at a laboratory local to each site. At Site 1 this was the Mycobacteriology Laboratory, Institut Pasteur du Cambodge, Phnom Penh (Lab 1). In Vietnam, the K74 Hospital Laboratory, Vinh Phuc Province (Lab 2), and the Centre for Tropical Medicine, Oxford Clinical Research Unit, Ho Chi Minh City (Lab 3) conducted testing for samples from Sites 2 and 3, respectively.

Following standard operating procedures at each laboratory, BAL samples underwent NaLC-NaOH decontamination, neutralisation with a phosphate buffer (PB), and centrifugation to produce a final pellet. For microscopy, slides were prepared from the final pellet, air dried, and then stained and examined for the presence of acid-fast bacilli using one of two methods, depending on the laboratory. At Labs 1 and 2, slides were stained with auramine dye using standard methods and examined using fluorescence microscopy. At Lab 3 slides were stained using the standard Ziehl-Neelsen procedure and examined using light microscopy.

Mycobacterial culture was performed on BAL samples at all three laboratories using liquid culture medium in mycobacteria growth indicator tubes (Bactec MGIT; BD Microbiology Systems, Cockeysvill, MD, USA) following standard operating procedures of the laboratory. Briefly, pellets were resuspended in PB then inoculated in liquid culture tubes and loaded into the BACTEC MGIT 960 system. Tubes were flagged as positive if growth was detected, and then subjected to a rapid immunochromatographic test (SD BIOLINE TB Ag MPT64 Rapid) targeting the MTBC specific antigen MPT64 to differentiate from MOTT. Cultures were considered MTBC negative if no growth was detected after six weeks or if MTBC was not confirmed by the immunochromatographic test.

PCR testing was carried out on BAL samples at Labs 1 and 3. For samples tested at Lab 1 prior to March 2019, an Xpert MTB-RIF assay kit (Cephid SAS, Sunnyvale, USA) was used, following the manufacturer’s instructions. This is a rapid cartridge-based assay using a heminested real-time PCR method to target the 81 base pair region of the rifampicin resistance determining region of the *rpoB* gene of the *M*. *tuberculosis* genome [46]. For samples tested at Lab 1 since March 2019, and all samples tested at Lab 3, an Xpert MTB/RIF Ultra assay kit (Cephid SAS, Sunnyvale, USA) was used, following the manufacturer’s instructions. This kit incorporates two different multi-copy amplification targets (IS*6110* and IS*1081*) along with fully nested nucleic acid amplification and melting temperature-based analysis to identify rifampicin resistance mutations in the *rpoB* gene [47].

### Statistical analysis

#### Target condition

A BLCM approach was used to model tests accuracy as a function of the obtained test results. The target condition for this BLCM was active pulmonary TB in bears with or without clinical signs consistent with TB. In definitions proposed for humans, a distinction is made between *M*. *tuberculosis* ‘infection’ which includes non-infectious latent, quiescent, or incipient states, and active TB ‘disease’, which is theoretically transmissible [15, 16]. Once infection advances to disease, increasing mycobacterial replication allows detection of radiographic changes and/or microbiological evidence of *M*. *tuberculosis*, which become more likely as disease progresses [48]. Human patients with active disease may be symptomatic or asymptomatic, however veterinary practice relies on observers recognising clinical signs, and thus the terms ‘clinical’ and ‘subclinical’ are appropriate to describe active TB with or without clinical signs, respectively, and our target condition of active pulmonary TB includes both.

#### Test targets

The three direct microbiological tests performed on BAL fluid target the presence of mycobacteria through microscopic evaluation for acid-fast bacilli (BAL microscopy), detection of *M*. *tuberculosis* DNA (BAL PCR), or growth and identification of live mycobacteria (BAL culture). Thoracic radiography allows evaluation of the thoracic cavity for typical radiographic manifestations of TB. The DPP VetTB serological assay targets antibody production in response to mycobacterial antigens. Thus, each test evaluated in this study is theoretically able to detect active pulmonary TB, where pulmonary disease is present with replicating mycobacteria and a simultaneous immune response, which may or may not be accompanied by clinical signs.

#### Bayesian Latent Class Model

We fitted a BLCM to estimate the sensitivity, specificity, predictive values, and likelihood ratios of five tests (BAL microscopy, BAL PCR, BAL culture, thoracic radiography, and the DPP VetTB assay) for detecting active pulmonary TB in sun bears and Asiatic black bears. To assess the impact of the DPP VetTB test positive threshold on diagnostic accuracy, we fitted the BLCM separately to the data stratified in two ways, according to the two different case definitions for the DPP VetTB test outlined above (i.e., with positive cases defined as either ‘strong’ reactions only, or ‘weak’ and/or ‘strong’ reactions). To account for a variation in the number of tests available for each population, the model included a different set of likelihood functions for each site. The accuracy of the three tests common to all three populations (BAL microscopy, BAL culture, and DPP VetTB) were therefore estimated with greater precision. The accuracy of the two remaining tests (BAL PCR and thoracic radiography) was estimated using results from two populations only. These results, however, contributed to estimating the other parameters.

The models were executed using JAGS (version 4.3.1) implemented via the R2jags package (version 0.7–1) using RStudio (version 2023.12.0) and R (version 4.2.0). The counts of the different combinations of positive and negative test results for the five tests under evaluation were assumed to follow a multinomial distribution. For the initial BLCM three important assumptions were made. We assumed that the five tests were conditionally independent, that the sensitivity (Se) and specificity (Sp) of the diagnostic tests were constant across the three populations, and that the disease prevalence varied across the populations. Under these assumptions, the number of degrees of freedom (n = 31) was higher than the 13 unknown parameters (the Se and Sp of five tests and the prevalence of disease in the three populations) to be estimated. The model was, therefore, identifiable.

Posterior inferences for each parameter were obtained from three Markov Chain Monte Carlo (MCMC) chains starting from different initial values, which were run for 21,000 iterations, after a burn in of 1,000 iterations. The impact of autocorrelation on the uncertainty of the estimates was assessed by measuring the effective sample size for each parameter using the effectiveSize function in R. For any given parameter, an effective sample size of 10,000 was deemed to be sufficient to describe the posterior distribution accurately. If the effective sample size was lower than 10,000, the number of iterations was increased until the targeted effective sample size was reached. The convergence and unimodality of the MCMC chains were assessed by visual inspection of the time series, autocorrelation plots and Gelman-Rubin plots, and by the posterior density distributions of each parameter, which were obtained using the ‘coda’ (version 0.19.4) and ‘mcmcplots’ (version 0.4.3) packages in R. Posterior distributions of each parameter were reported as medians and corresponding 95% Bayesian credible intervals (BCI).

#### Priors

Because there is no information in the literature reporting the accuracy of any tests for diagnosing TB in bears and the TB prevalence in the study populations was unknown, a uniform prior distribution (*Beta* (1, 1)) was used for all parameters of interest in the initial model. Since false positive results for *M*. *tuberculosis* detection by PCR and culture are unlikely [49, 50], we performed a first sensitivity analysis on the initial model using weakly informative priors for specificity of BAL PCR and BAL culture. Under the assumption that the specificity of those tests was unlikely to be lower than 90%, a uniform distribution between 0.9 and 1.0 was chosen to inform the specificity of both tests for the sensitivity analysis.

#### Assumption of conditional independence

For the three microbiological tests (BAL microscopy, BAL PCR, and BAL culture), the assumption of conditional independence from the true health status of the bear was questionable since each test relies on the detection of mycobacteria or mycobacterial DNA and they were all performed on the same BAL specimen. Therefore, as a second sensitivity analysis, BLCMs allowing for pairwise conditional dependence between these three tests were run. This was achieved by adding pairwise covariance terms between their sensitivities and specificities. The prior distributions of the covariance parameters were defined as follows [51]:

Covp∼dunif((Se1−1)*(1−Se2),min(Se1,Se2)−Se1*Se2)

Covn∼dunif((Sp1−1)*(1−Sp2),min(Sp1,Sp2)−Sp1*Sp2)

where Covp represents the covariance of two tests among the truly diseased (i.e., bears with TB), and Covn represents the covariance of two tests in truly healthy bears (i.e., bears without TB). Both Covp and Covn were assumed to follow uniform distributions limited by their natural limits as described by Dendukuri and Joseph [52].

#### Scenario-based evaluation of test application

Positive and negative predictive values were computed directly within Jags using results generated from the BLCM. Because the true prevalence of TB was unknown at the study sites, and to illustrate the impact of population prevalence on predictive values of tests under different scenarios, we computed the values at two hypothetical disease prevalence levels. We used a 5% prevalence to demonstrate predictive values when using the tests to monitor a population where TB is rare or not expected, and 50% prevalence used to demonstrate predictive values when using the tests to diagnose TB in a population where test subjects are considered at high risk of having TB.

We calculated positive (and negative) likelihood ratios to illustrate the value of a positive (and negative) test result irrespective of the TB prevalence. The positive and negative likelihood ratios were computed directly within Jags using results generated from the BLCM model. Positive and negative likelihood ratios were used to demonstrate how a test result might update the clinician’s prior belief regarding the disease status of a given animal (i.e., the diagnostic value added by the test) depending on the pre-test level of clinical suspicion. We combined computed test likelihood ratios with five hypothetical pre-test disease probabilities (0.025, 0.1, 0.25, 0.5, 0.75) to calculate post-test disease probabilities, according to the following equations:

$$Pre-test\;odds=\frac{pre-test\;probability}{\left(1-pre-test\;probability\right)}$$

Post−testodds=pre−testoddsxlikelihoodratio

$$Post-test\;probability=\frac{post-test\;odds}{1+post-test\;odds}$$

## Results

### Study population

**Fig 1:**
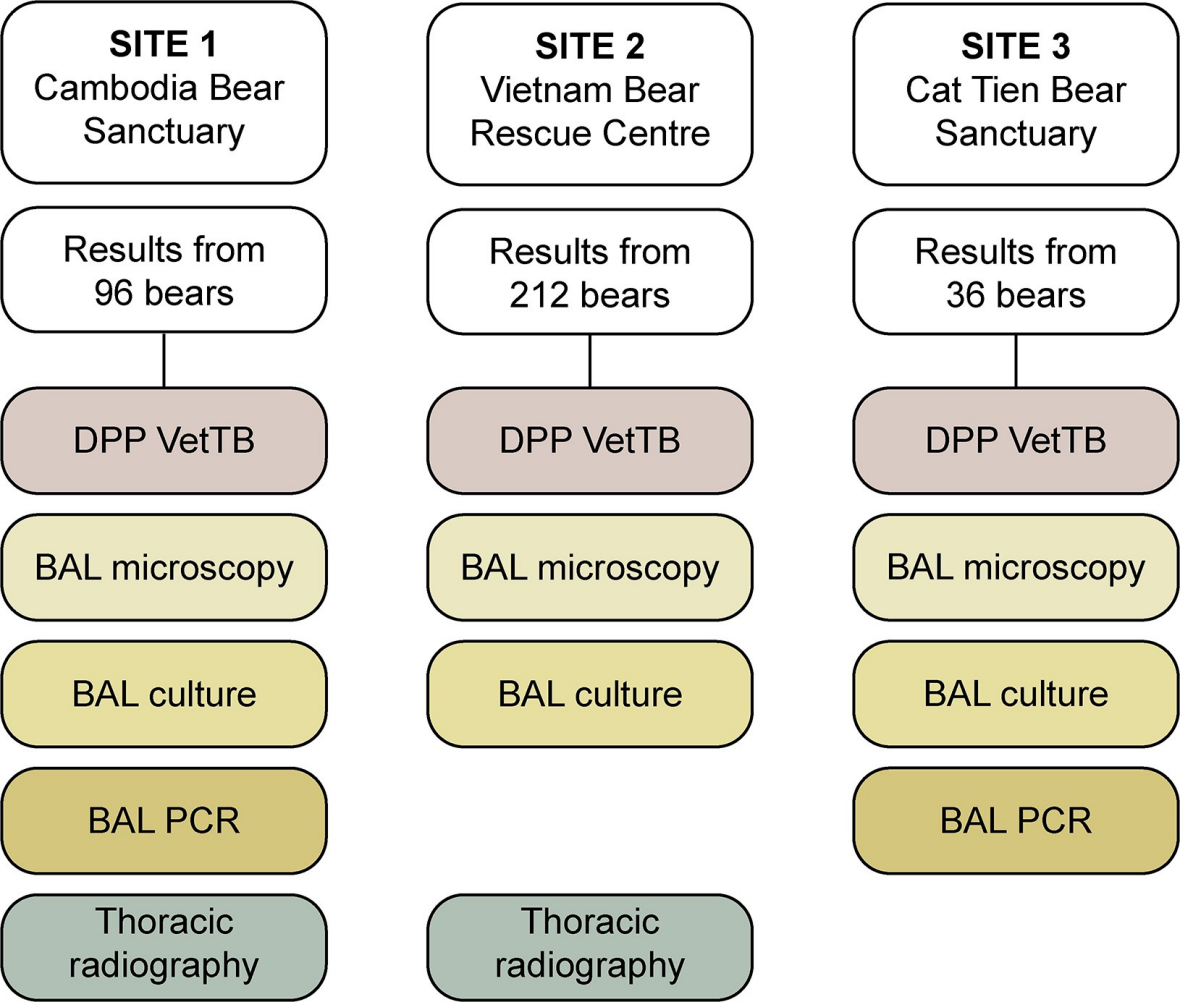
Study population showing number of available test results by site. Flow chart indicates the total number of bears included in the study from each site, and the combination of tests used at that site to screen bears for pulmonary tuberculosis. Tests included a serological assay (DPP VetTB), thoracic radiography, and microscopy, PCR (Xpert MTB/RIF or MTB/RIF Ultra) and culture of bronchoalveolar lavage (BAL) samples.

At Site 1, bears that did not have results for all five tests conducted at a single time point were excluded, leaving results from 96 out of 162 bears at risk available (Fig 1). At Site 2, PCR was not routinely requested and thus results were available for four tests (excluding BAL PCR) from 212 of 261 bears at risk. Radiography was not consistently available at Site 3 and therefore results were included for four tests conducted at that site (excluding thoracic radiography) for 36 of 55 bears at risk. Baseline demographic information for bears included in the study is shown in Table 2.

**Table 2:** Distribution by species, sex, age, and location of 344 captive bears used to estimate the accuracy of five tests to diagnose pulmonary tuberculosis.

		Site 1 Cambodia Bear Sanctuary (n = 96)	Site 2 Vietnam Bear Rescue Centre (n = 212)	Site 3 Cat Tien Bear Sanctuary (n = 36)
**Species**	Asiatic black bear	18	203	28
	Sun bear	78	9	8
**Sex**	Male	36	93	11
	Female	60	119	25
**Age**	<10 years	23	27	2
	10–20 years	50	146	25
	>20 years	23	39	9

### Test results combinations

**Fig 2:**
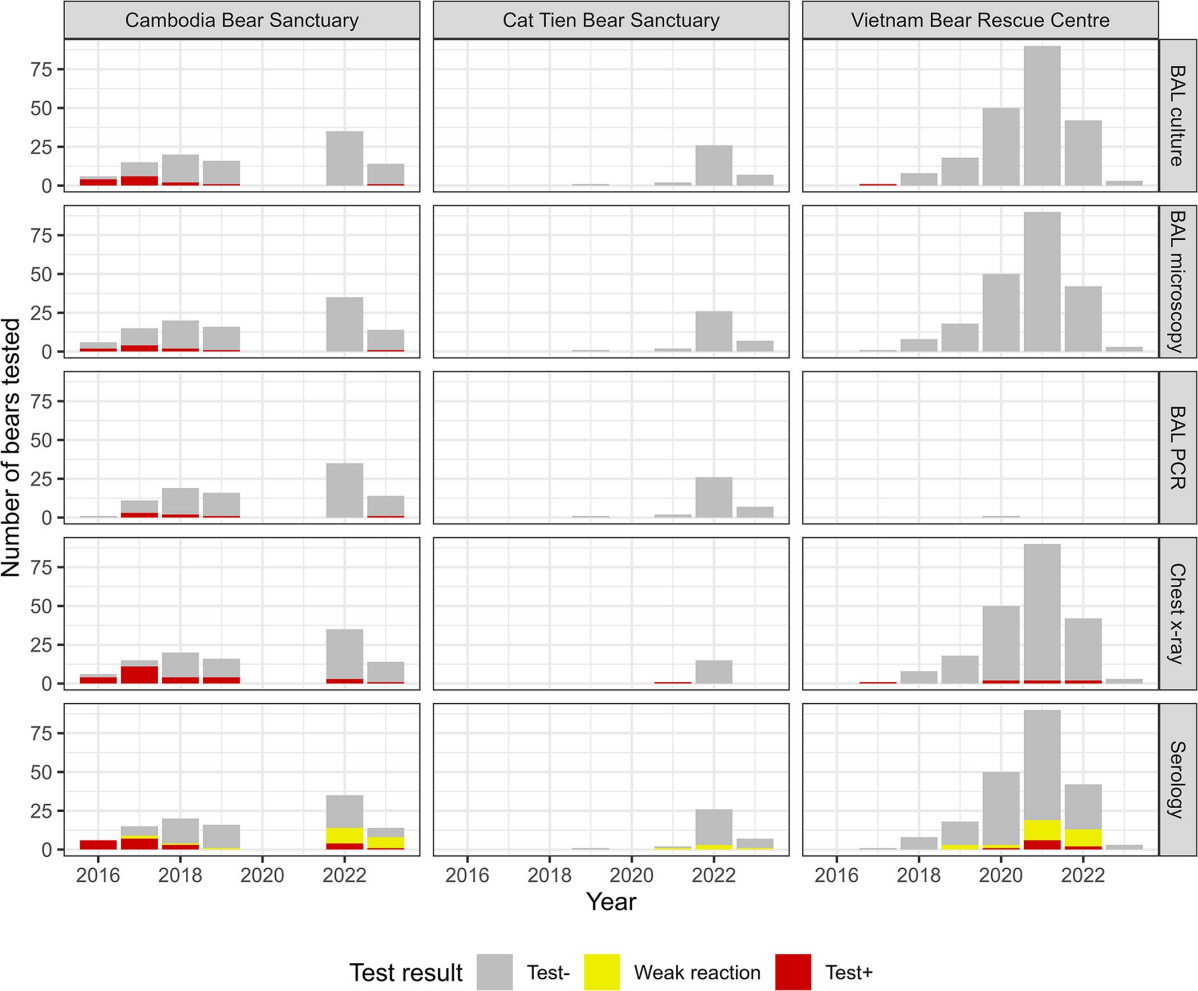
Distribution by year and site of results from five tests applied to 344 captive bears in Cambodia and Vietnam. Tests included thoracic radiography (chest x-ray), serology (DPP VetTB assay, with two definitions of positive depending on the strength of the reaction line), and microscopy, PCR, and culture of bronchoalveolar lavage (BAL) samples.

Test results obtained from 344 bears between 2016–2023 are shown in Fig 2, including those undergoing five tests at Site 1, and two different combinations of four tests at Sites 2 and 3, and according to two different case definitions for a positive serology test. Cross classified results showing the number of bears at each site with given test results profiles, are fully reported in the data repository files. The strength and line position of all reactive DPP VetTB serological assays included in the BLCA, along with those from any additional culture confirmed TB cases, are available in S1 Table.

### BLCMs and sensitivity analyses

**Fig 3:**
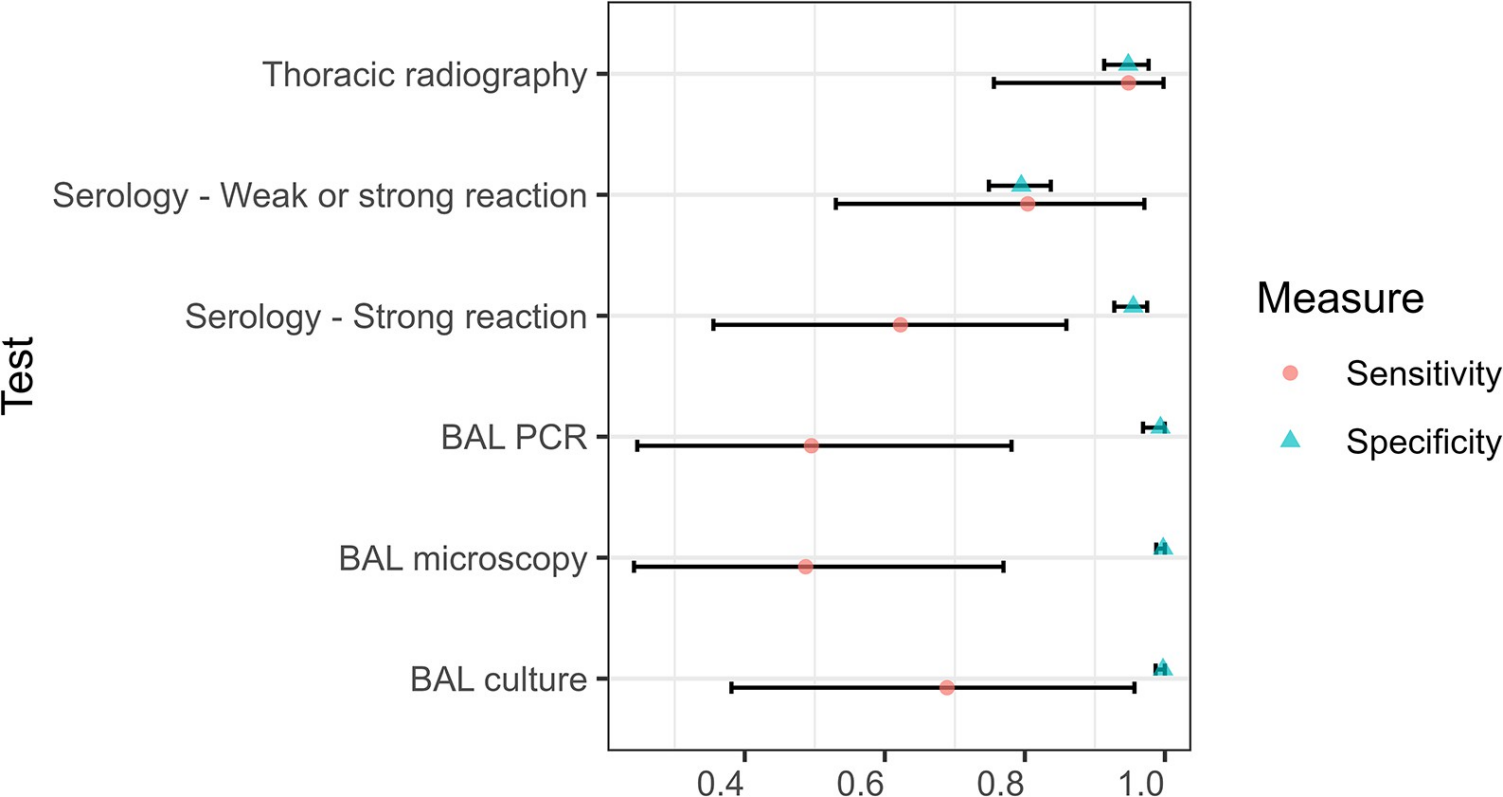
Sensitivity and specificity of five tests used to diagnose pulmonary tuberculosis in captive bears in Cambodia and Vietnam. Tests included thoracic radiography, serology (DPP VetTB assay, with two definitions of positive depending on the strength of the reaction line), and microscopy, PCR, and culture of bronchoalveolar lavage (BAL) samples. Median (points) and 95% Bayesian credible intervals (bars) are shown for estimates derived from a Bayesian latent class model assuming conditional dependency between BAL microscopy and BAL PCR.

**Fig 4:**
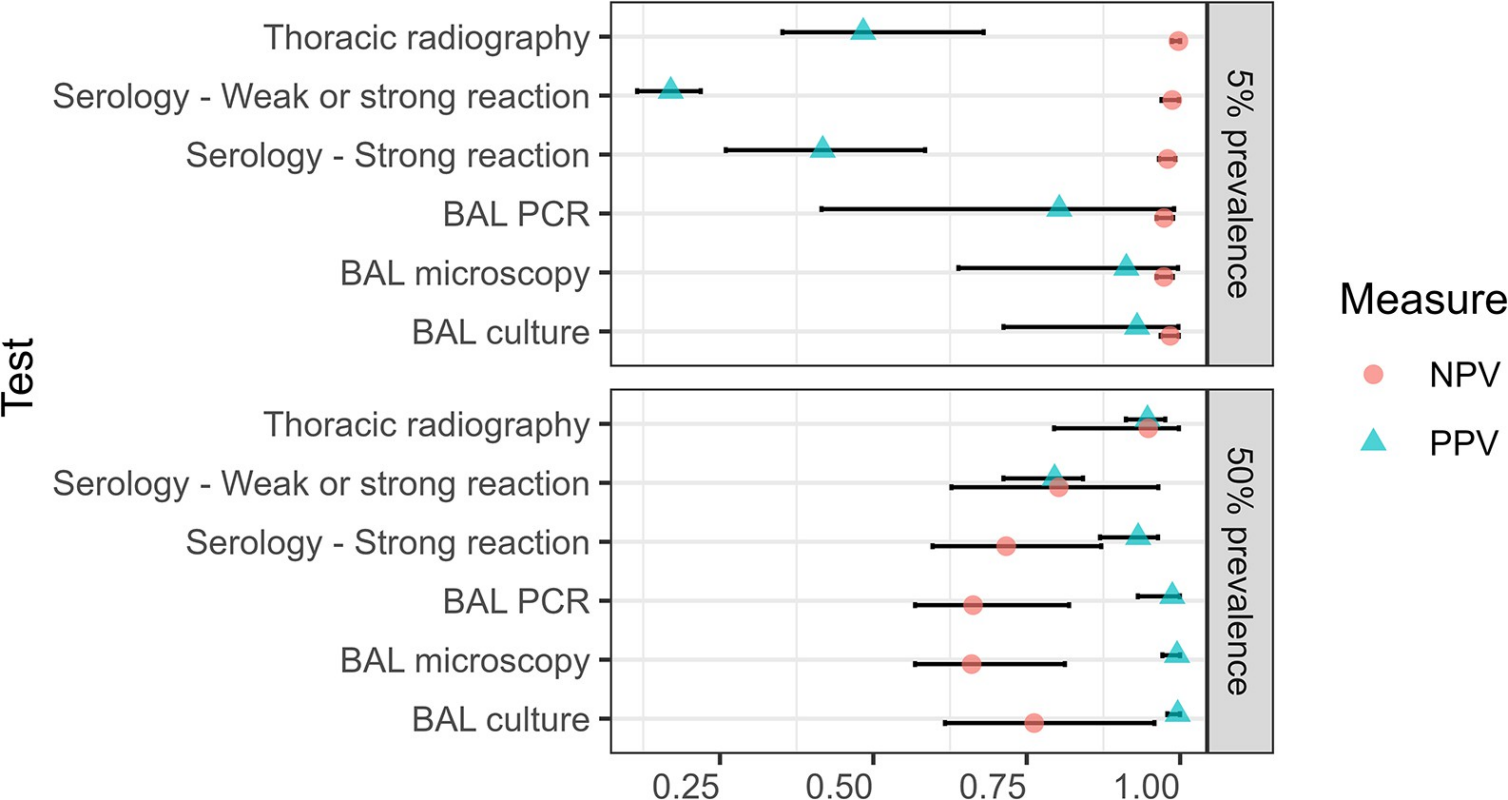
Positive (PPV) and negative (NPV) predictive values of five tests used to diagnose pulmonary tuberculosis in 344 captive bears in Cambodia and Vietnam. Tests included thoracic radiography, serology (DPP VetTB assay, with two definitions of positive depending on the strength of the reaction line), and microscopy, PCR, and culture of bronchoalveolar lavage (BAL) samples. Median (points) and 95% Bayesian credible intervals (bars) are shown for estimates calculated for two disease prevalence scenarios using a Bayesian latent class model assuming conditional dependence between BAL microscopy and BAL PCR.

**Fig 5:**
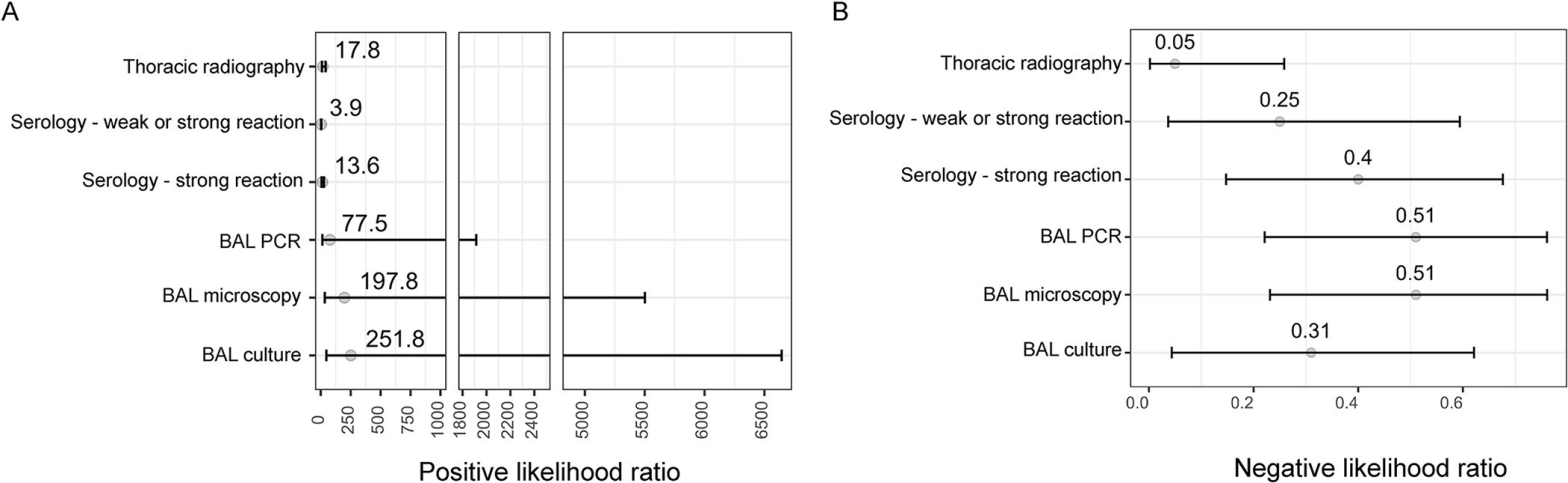
Positive (A) and negative (B) likelihood ratios of five tests used to diagnose pulmonary tuberculosis in 344 captive bears in Cambodia and Vietnam. Tests included thoracic radiography, serology (DPP VetTB assay, with two definitions of positive depending on the strength of the reaction line), and microscopy, PCR, and culture of bronchoalveolar lavage (BAL) samples. Median (points) and 95% Bayesian credible intervals (bars) are shown for estimates derived from a Bayesian latent class model assuming conditional dependence between BAL microscopy and BAL PCR.

Estimates of test accuracy were obtained from the initial BLCM using uniform priors and assuming conditional independence between all tests (Table 3). Adding weakly informative priors for the specificities of BAL culture and BAL PCR, as well as investigating pairwise conditional dependency between BAL culture and BAL microscopy and between BAL culture and BAL PCR had little effect on the posterior distributions for all test parameters (all sensitivity analyses are fully reported in the data repository files). On the other hand, a relatively large positive covariance was observed between BAL microscopy and BAL PCR, suggesting these tests were conditionally dependent among the truly diseased animals. This latter, final, BLCM allowing for conditional dependence between BAL microscopy and BAL PCR was therefore retained for presentation of results (Table 3). Finally, two sets of accuracy results for serology were computed, reflecting the different case definitions for the serological test: one including only strong reactions as positive and the other including weak or strong reactions as positive. In the final model, changing the definition of positive on the serological test had little effect on the parameters for the other tests, thus we elected to use the definition that treated only strong reactions as positive to derive the accuracy parameters for the remaining tests. Results from the final BLCM are presented in Figs 3–5.

**Table 3:** Posterior distributions derived from two Bayesian latent class models (BLCMs) to estimate the sensitivity and specificity of five tests to diagnose pulmonary tuberculosis in 344 captive bears in Cambodia and Vietnam.

Test Parameter	Posterior distributions median (95% Bayesian credible interval)	
	Initial BLCM assuming conditional independency between the tests	Final BLCM allowing for dependency between BAL microscopy and BAL PCR
**BAL microscopy**		
Sensitivity	0.56 (0.28, 0.83)	0.49 (0.24, 0.77)
Specificity	0.997 (0.99, 1.00)	0.998 (0.99, 1.00)
**BAL PCR**		
Sensitivity	0.59 (0.31, 0.86)	0.50 (0.25, 0.78)
Specificity	0.99 (0.97, 1.00)	0.99 (0.97, 1.00)
**BAL culture**		
Sensitivity	0.81 (0.51, 0.99)	0.69 (0.38, 0.96)
Specificity	0.996 (0.99, 1.00)	0.997 (0.99, 1.00)
**Thoracic radiography**		
Sensitivity	0.93 (0.75, 0.998)	0.95 (0.76, 0.998)
Specificity	0.94 (0.91, 0.96)	0.95 (0.91, 0.98)
**DPP (positive: strong reactions only)**		
Sensitivity	0.65 (0.38, 0.88)	0.62 (0.36, 0.86)
Specificity	0.95 (0.92, 0.97)	0.96 (0.93, 0.98)
**DPP (positive: strong and weak reactions)**		
Sensitivity	0.82 (0.55, 0.98)	0.80 (0.53, 0.97)
Specificity	0.79 (0.74, 0.83)	0.80 (0.75, 0.84)
**Covp**	n/a	0.18 (0.08, 0.24)
**Covn**	n/a	0.001 (0, 0.007)

Prior distribution for all parameters was β (1;1)

BAL: bronchoalveolar lavage

DPP: Dual path platform VetTB serological assay

n/a: not applicable

Covp: covariance between the sensitivity of the tests among TB positive bears

Covn: covariance between the specificity of the tests among TB negative bears

### Tests accuracy

The microbiological tests (BAL microscopy, BAL PCR and BAL culture) each had near perfect specificity, however the sensitivity estimates for these tests were imprecise (Table 3 and Fig 3). Including weak DPP VetTB test reactions as positive increased the sensitivity of the serological test from 0.62 (95% BCI: 0.36, 0.86) to 0.80 (95% BCI: 0.53, 0.97), however this was at the cost of specificity, which reduced from 0.96 (95% BCI: 0.93, 0.98) to 0.80 (95% BCI: 0.75, 0.84). Thoracic radiography had the highest sensitivity point estimate (0.95; 95% BCI: 0.76, 0.998) however the specificity for this test (0.95; 95% BCI: 0.91, 0.98) was lower than for the microbiological tests (all ≥ 0.99) and a strongly reactive DPP VetTB test (0.96; 95% BCI: 0.93, 0.98).

### Predictive values

Predictive values were computed for two different theoretical prevalence levels, using results generated by the final model and including the two different definitions for a positive DPP VetTB test (Fig 4). When the prevalence was set at 5%, the positive predictive values (PPV) of tests ranged from a low of 0.17 (95% BCI: 0.12, 0.22) for the DPP VetTB serological test when weak reactions were included as positive, to a high of 0.93 (95% BCI: 0.71, 0.997) for BAL culture. At a higher theoretical prevalence of 50%, the PPV of all tests increased to above 0.9, apart from that of the DPP VetTB test with weak reactions included (PPV = 0.80; 95% BCI: 0.71, 0.84). The negative predictive values (NPV) of all tests under evaluation were high at a 5% theoretical prevalence level. When the prevalence was set to the higher level of 50%, the NPV of each test reduced, although the point estimate for thoracic radiography remained above 0.9 (NPV = 0.95; 95% BCI: 0.80, 0.998).

### Likelihood ratios

Positive (LR+) and negative (LR-) likelihood ratios were computed using results generated by the final model, and for each case definition for positive DPP VetTB (Fig 5). The LR+ of all the tests under evaluation were greater than 10 (that is, the probability of a bear with a positive test result having TB is at least 10 times greater than a bear with a negative test result), except for the DPP VetTB test when weak reactions were treated as positive (LR+ = 3.89; 95% BCI: 2.47, 5.32). Only one test, thoracic radiography, had an LR- point estimate of less than 0.1 (that is, the level at which a negative test result is at least 10 times more common in bears without TB than those with TB; LR- = 0.05; 95% BCI: 0.002, 0.26).

**Fig 6:**
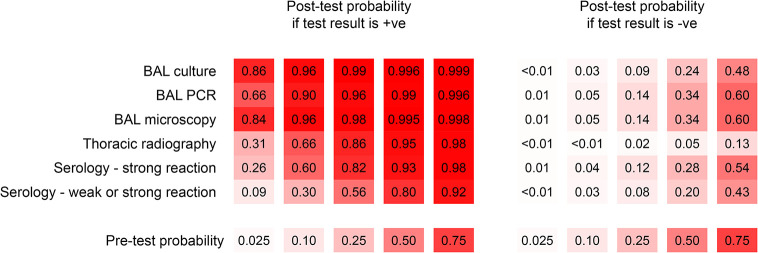
Post-test disease probabilities for positive and negative test results from five tests used to diagnose pulmonary tuberculosis in 344 captive bears in Cambodia and Vietnam. Tests included thoracic radiography, serology (DPP VetTB assay, with two definitions of positive depending on the strength of the reaction line), and microscopy, PCR, and culture of bronchoalveolar lavage (BAL) samples. Post-test disease probabilities were calculated for each test at five different pre-test probability levels and using results derived from a Bayesian latent class model assuming conditional dependence between BAL microscopy and BAL PCR. Increasing colour gradient (0–100%) is used to represent increasing probability of disease (0–1).

Post-test probabilities derived from the computed positive and negative likelihood ratios are shown in Fig 6. At pre-test probabilities (reflecting theoretical levels of pre-test clinical suspicion) above 0.025, a negative thoracic radiographic study had the most influence on the post-test probability of disease compared with any other test. At a relatively high pre-test probability (0.75), the post-test probability of disease ranged within 0.43–0.60 for a negative result on any of the BAL microbiological tests or the DPP VetTB serological test, whereas it was 0.13 based on a negative radiographic study. When looking at the effect of positive results on the probability of disease, a positive result on any of the BAL microbiological tests when pre-test probability was low translated to a relatively high post-test probability of disease, and this became almost certain (≥ 0.99) if the pre-test probability was already 0.5 or above. In contrast, after a positive DPP VetTB test (including weak reactions) the probability of disease was 0.09 when clinical suspicion was very low (0.025 pre-test probability) and rose to above 0.90 only when clinical suspicion was already very high (i.e. 0.75 pre-test probability).

## Discussion

Using BLCA and data from 344 bears, we provide the first insights into the diagnostic performance of five tests for ante-mortem diagnosis of pulmonary TB in bears in sanctuary settings. Our results confirm the high specificity of microbiological tests and identify thoracic radiography as the only test with point estimates above 90% for both sensitivity and specificity. Further, we highlight the trade-off that occurs between sensitivity and specificity when using two different visual cut-offs for a commercially available serological test. We use predictive values, at different prevalence levels, and likelihood ratios, at different probabilities of disease, to highlight the impact of context when interpreting test results. Our findings reinforce the need to approach TB testing in these species strategically, considering the consequences test results (including misclassifications) may have for individual animals and the risk of disease transmission.

BLCA is the recommended statistical approach to diagnostic accuracy studies when a suitable standard reference test does not exist [43, 44, 51, 53], as is the case when evaluating ante-mortem tests for TB [54–56]. While BLCMs may perform better with the addition of prior information, the lack of previous studies investigating the accuracy of the tests in bear species precluded robust priors, and our sensitivity analyses demonstrated minimal impact on posterior results when adding priors for the highly specific microbiological tests. We investigated conditional dependency between the three microbiological tests, given each test directly detects mycobacteria in the same respiratory tract specimen [57, 58]. A positive covariance was found between BAL microscopy and PCR suggesting that both tests were dependent conditionally on the true TB status of bears and this may have affected the sensitivity estimates for these tests. The positive covariance term included in the model allows for the possibility that the same misclassification error may occur for both tests among the truly diseased bears, with bears classified as having active pulmonary TB on BAL microscopy also having a higher probability of being classified as truly diseased on BAL PCR. Also, it may be possible that both tests could miss the same truly positive bear given that both tests have imperfect sensitivities (around 50%) indicating that they are both likely to misclassify a similar proportion of bears with active pulmonary TB.

Our results show that thoracic radiography had a relatively high sensitivity (95%) for detecting pulmonary TB in these bear species, although we note the imprecision of our estimates owing to the small number of positive test results in the study population. This sensitivity estimate correlates with several human studies [25, 54, 59, 60], with veterinary studies lacking as the size of species commonly screened for TB (e.g., elephants and livestock) limits the use of radiography. The relatively high sensitivity estimate in our study could be explained by multiple factors, including advanced disease at the time of testing (we used the most recent test if animals had serial testing), and our broad case definition for thoracic radiography which was based on clinical interpretation of radiographs with any abnormalities consistent with TB, an approach in line with chest radiograph accuracy studies for human TB [61]. The estimated specificity of thoracic radiography was also relatively high (95%), in contrast to some human studies [24, 62], and perhaps related to the lack of other pulmonary conditions in the bear population. However, in a low prevalence setting, the risk of falsely ascribing radiographic changes to TB is emphasised, with a declining positive predictive value for this test. Overall, despite infrastructure and cost considerations, thoracic radiography is a useful screening tool for pulmonary TB in bears in a rescue centre context, given the in-house convenience, options for specialist input via teleradiology, and the test accuracy parameter results.

When weak reactions were included as positive, the DPP VetTB serological test was the only other test in our study with a point estimate for sensitivity at or above 80%, dropping to 62% when only strong reactions were included as positive. These results illustrate the trade-off that occurs when the stringency of a positive cut-off is changed; sensitivity (the probability that diseased animals test positive) is improved if weak reactions are included as positive, however at a cost to specificity (the probability that healthy animals test negative), which dropped from 96% to 80%. Thus, when treating weak reactions as positive, the risk of false negatives (missing diseased individuals) is reduced, however the increased risk of false positives (including healthy individuals as ‘diseased’) needs to be accounted for. Conversely, treating weak reactions as negative protects against false positives, yet lowers sensitivity and thereby increases the risk that missed diseased animals (false negative results) will propagate spread. The decision on how to treat a weak reaction should be flexible, depending on the disease management strategy (prevention of spread versus preservation of life) and the consequences of misclassification (e.g., false positives resulting in unnecessary euthanasia, indefinite isolation, or potentially dangerous and/or expensive treatment; false negatives resulting in disease transmission).

Our accuracy results for the DPP VetTB serological test were lower than those reported in the target species. In elephants, 100% specificity (95% CI: 96.8, 100) and 100% sensitivity (95% CI: 84, 100) were reported when reactions to Line 2 were considered, reflecting the serodominance of antibodies to CFP10/ESAT in *M*. *tuberculosis* infected elephants [34]. While reactions at Line 1 predominate in certain *M*. *bovis* infected animals [37, 41, 42], when seen alone in elephants they may indicate cross reactivity to a homologous protein expressed by MOTT or other environmental bacteria, or possibly to rheumatoid factor in arthritic individuals [34, 63]. However, given reactions at Line 1 alone are rare but possible in elephants with TB [42, 64], the test instructions recommend interpreting a Line 1 reaction in elephants as “TB or mycobacteriosis”. In this study, we considered a reaction at either or both test lines as positive, given evidence that a reaction at Line 1 alone is possible in bears with confirmed TB (S1 Table), however this likely contributed to the reduced specificity results for this test. Given the detection of clinically important MOTT in the respiratory tract of other TB susceptible species [65], and the relatively high number of weak Line 1 reactions in our study, further investigation into cross-reactivity and the potential contribution of MOTT and/or chronic inflammatory conditions on this test in bear species is warranted. There is a lack of studies investigating the accuracy of the test when used off-label to detect antibody responses to *M*. *tuberculosis* specifically in non-target species, although one infected black rhino (*Diceros bicornis*) produced responses over 12 years prior to death with TB caused by *M*. *tuberculosis* [66]. A MAPIA-based evaluation of other, novel, *M*. *tuberculosis* antigens that might be preferentially recognised by bear species would expand knowledge of differential host antibody reactivity profiles.

Culture of respiratory samples remains the definitive ante-mortem test to confirm pulmonary TB in humans and other animals, despite the challenge of low sensitivity and the lag time for results [67–70]. The imprecision around our sensitivity estimates precludes strong conclusions, however estimated sensitivity could be as low as 38% for culture of BAL samples, and even lower for microscopy (24%) and PCR (25%). To optimise ante-mortem recovery of mycobacteria from the respiratory tract, pooling of multiple sputum samples is recommended in humans [71], as is a triple trunk wash sampling protocol in elephants [72], however, while BAL allows deeper respiratory tract sampling, repeat sampling is constrained by the need for general anaesthesia. The lower detection threshold of mycobacterial culture (1–10 Colony Forming Units (CFU)/ml) compared with PCR (15–143 CFU/ml) and microscopy (10,000–100,000 CFU/ml) [47, 73]) likely explains the potentially superior diagnostic sensitivity of culture demonstrated in our results and underscores the importance of waiting the additional time for culture results if the more timely direct tests are negative. When the prevalence was set to 50%, the negative predictive values of the BAL microbiological tests all reduced. In practice, this means caution should be exercised if disease is expected and these tests are negative, whereas a negative radiographic study would be useful to rule active pulmonary TB out, due to its higher negative predictive value under this scenario.

As expected, our results confirm the high specificity of the three direct microbiological tests, with point estimates for BAL microscopy, PCR, and culture all approaching 100%. False positive results due to non-viable organisms are possible on PCR, however this would not be expected in this bear population, with no treatment undertaken. Microscopy is prone to lower specificity due to its inability to distinguish between different species of mycobacteria, particularly when the collection method, such as sputum production in humans and trunk wash in elephants, increases the chance of contamination with MOTT from the upper respiratory tract. This is bypassed when using BAL and may have contributed to the comparable specificity of all three mycobacterial tests in our study, which underscores the utility of these tests for confirming active disease. In contrast, even the seemingly reasonable specificities of thoracic radiography (95%) and a strongly reactive serological test (96%) equate to a risk that around one in 20 positive test results is likely to be false. Particularly in low prevalence settings, where the positive predictive value of these tests is reduced (Fig 4), caution is recommended if drastic action is to be taken in response to positive test results.

Along with prevalence, other contextual factors may influence how tests are used and their results interpreted, as demonstrated by the application of likelihood ratios at different pre-test probabilities of disease. The only test with a positive likelihood ratio below 10 (the level above which a positive result is considered strong evidence for disease being present) was the DPP VetTB serological test when weak reactions were included as positive, confirming the improved diagnostic value of a strongly positive result on this test. The very high positive likelihood ratios of the microbiological tests update the post-test probability of disease to high, regardless of the pre-test probability, confirming that positive results on these tests should not be ignored, regardless of clinical suspicion level. In contrast, a weakly positive serology result in a bear with a low pre-test probability of disease adds minimal diagnostic value, barely updating the post-test probability of disease. However, if clinical suspicion was already high (0.75), the same result would update the post-test probability to above 0.9, reflecting enhanced usefulness of this test when disease is likely. While we acknowledge the imprecise sensitivity estimates led to wide credible intervals for the negative likelihood ratio estimates, our results highlight thoracic radiography as the only test with a negative likelihood ratio estimated to be less than 0.1 (the level below which a negative result is considered as strong evidence that disease is unlikely). Even at 0.75 pre-test probability, a negative thoracic radiographic study reduced the post-test probability of pulmonary TB to just 0.13, demonstrating its utility for updating clinical suspicion away from pulmonary TB if typical radiographic changes are not seen. In contrast, a negative BAL culture or a lack of strong response on the serological test left an around 1 in 2 chance of disease in fact being present.

This study had several limitations, notably the low number of positive results, as well as pooling of results across the two bear species, which led to imprecision around sensitivity estimates, and prevented analysis of species as a confounder. As there may be species-specific immune responses to *M*. *tuberculosis* antigens, future studies should consider the effect of species on serological testing. The low number of cases also precluded analysis of clinical status at the time of testing, however we used predictive values and likelihood ratios to explore the application of our results under different clinical scenarios. Systematic error in the form of observer bias and misclassification is also possible as we relied on subjective interpretation of the visible strength of test lines on the DPP VetTB assay and thoracic radiograph findings. It is possible some of the weak DPP VetTB reactions would be negative if interpreted by an optical density reader using pre-determined cut-off values, thereby improving the estimated specificity of the test. However, given this equipment is not readily available to clinicians in the field, we considered it reasonable to use data from visual evaluations. Both the serological test and thoracic radiographs were classified by clinicians with prior knowledge of the bear’s health and potential exposure status, which also introduces the possibility of variable and/or biased interpretations. Further, given the likelihood of being radiograph-positive is linked to the progression of disease, it is possible that we did not account for conditional dependence between this and the direct tests [57]. We did not account for the improved sensitivity of the newer PCR test [47] used at Lab 3 and adopted at Lab 1 during the study period, however considered this acceptable given it was unavoidable and that there was already considerable imprecision around our sensitivity estimates. Likewise, the radiographic equipment used at Sites 1 and 2 was not identical, however this was also unavoidable, and the quality of images was considered consistent. Finally, it is possible that a detectable immune response either pre-empts our target condition of active pulmonary TB, as shown in elephants [64] and rhinoceros [66], or occurs due to mycobacterial infection at extra-thoracic sites. This raises the possibility that bears that were positive on DPP VetTB and negative on the tests for pulmonary disease either had extra-pulmonary TB or may go on to develop pulmonary TB at some time in the future. Prospective studies testing serum from at-risk bears at regular time intervals prior to the potential development of TB are needed before firm conclusions can be drawn on immunoreactivity to key MTBC antigens in these species.

## Conclusion

This study provides insights into the accuracy and application of tests available for the diagnosis of TB in Southeast Asian bear sanctuaries. Along with health and welfare consequences for captive bears, the presence of TB threatens conservation-focussed release programs and is a transmission risk for in-contact humans and other susceptible species. Detection of active cases as early as possible is key to control and relies on informed selection and interpretation of diagnostic tests. A clear disease management strategy that considers the potential consequences of misclassification will also determine how tests are applied and results interpreted. Taken together our results support the use of thoracic radiography and the DPP VetTB serological test as screening tests, with confirmation by direct microbiological tests, particularly when positive results are unexpected and/or when a test and cull strategy is being applied. We provide clarification around interpreting line strength on the DPP VetTB test, with the high specificity of a strong reaction highlighting this as a convenient patient-side test. However, we caution against ruling in disease based on weak reactions on this test alone, and recommend that results are confirmed with another test and that uncertainty around reaction strength and visibility is considered in the light of the reason for testing, the testing context, and the consequences of misclassification.

## Supporting information

S1 Table Seroreactivity pattern description for all DPP VetTB test results. (PDF)
