# Supplementary material for: Bayesian accuracy estimates for diagnostic tests to detect tuberculosis in captive sun bears (Helarctos malayanus) and Asiatic black bears (Ursus thibetanus) in Cambodia and Vietnam
Source: PLoS One. 2024 Nov 13;19(11):e0313007. doi: 10.1371/journal.pone.0313007 (PMC11560021; doi:10.1371/journal.pone.0313007)
Supplement: S1 Table — (PDF) [file pone.0313007.s001.pdf]

**S1 Table. Seroreactivity pattern description for all DPP VetTB serological test results included the Bayesian latent class analysis (BLCA) and those conducted on serum from culture-confirmed tuberculosis (TB) positive bears at Sites 1 and 2 between February 2016 and March 2023.**

| Line 1 MBP83 | Line 2 CFP10/ESAT6 | Number of results included in BLCA | Number of results from known TB positive bears* |
|---|---|---|---|
| Strong | Strong | 4 | 7 |
| Strong | Weak | 6 | 2 |
| Strong | None | 11 | 3 |
| Weak | Strong | 2 | 1 |
| Weak | Weak | 13 | 2 |
| Weak | None | 32 | 1 |
| None | Strong | 6 | 6 |
| None | Weak | 10 | 1 |
| None | None | 260 | 5 |
| | **Total** | **344** | **28** |

*not all DPP VetTB results from culture-confirmed tuberculosis positive bears were available for inclusion in the BLCA due to not having an eligible full set of results
